# Recovery beyond ejection fraction after takotsubo syndrome: a multidimensional framework for risk-enriched follow-up

**DOI:** 10.3389/fcvm.2026.1960498

**Published:** 2026-09-24

**Authors:** Shuxia Liang, Lu Xu, Zheng Hu

**Affiliations:** 1 Emergency Department, The Second Hospital of Hebei Medical University, Shijiazhuang, Hebei, China; 2 Department of Cardiology, The Second Hospital of Hebei Medical University, Shijiazhuang, Hebei, China

**Keywords:** cardiac rehabilitation, cardiovascular magnetic resonance, left ventricular ejection fraction, multidimensional recovery, stress cardiomyopathy, takotsubo syndrome

## Abstract

Takotsubo syndrome (TTS) is defined by transient regional ventricular dysfunction, and normalization of left ventricular ejection fraction (LVEF) remains the conventional marker of recovery. This endpoint is clinically useful but does not capture the tempo or full clinical breadth of convalescence. Registry and multimodality studies indicate that ventricular recovery may be delayed or asynchronous; some survivors report persistent symptoms or functional limitation, whereas others have subclinical abnormalities in myocardial deformation, tissue characteristics, energetics, microvascular function, or inflammation. The clinical meaning and causal attribution of many subclinical findings remain uncertain. This State-of-the-Art Review examines how LVEF became the default recovery endpoint and synthesizes evidence across mechanical, clinical/functional, and biological/subclinical domains. We propose a staged framework in which recovery trajectory, persistent symptoms, and severe cardiovascular complications identify unresolved cardiovascular recovery concerns, whereas malignancy, neurological disease, physical triggers, and major systemic comorbidity primarily indicate competing risk and a need for coordinated care. Strain imaging, cardiovascular magnetic resonance, cardiopulmonary exercise testing, and structured patient-reported outcomes should be selected only when they address a defined clinical question. Management remains indication-driven, with complication surveillance and targeted rehabilitation or psychological care when limitations persist. The framework organizes clinical reasoning and trial design but is not a validated risk score or management algorithm. Prospective studies should define temporal recovery stages, establish a pragmatic core recovery set, and test whether adjunctive assessments or recovery-directed interventions improve clinically meaningful outcomes.

## 1 Introduction

Takotsubo syndrome (TTS) is an acute heart failure syndrome that commonly presents as an acute coronary syndrome mimic. Chest pain, electrocardiographic changes, biomarker release, and transient regional ventricular dysfunction frequently prompt urgent coronary assessment, while the absence of a culprit lesion and the characteristic pattern of wall-motion abnormality establish the diagnosis (1). This acute resemblance is clinically important because TTS is not a benign diagnostic detour. In the International Takotsubo Registry, severe in-hospital complications occurred at rates comparable with acute coronary syndrome (2). Contemporary registry data also show a changing case mix, with more physical triggers, more cardiogenic shock, and higher early mortality (3). A Swedish nationwide analysis placed 30-day mortality below that of ST-segment elevation myocardial infarction but above that of non-ST-segment elevation myocardial infarction (4).

The language of reversibility has nevertheless shaped post-acute care. In routine practice, recovery is often declared when left ventricular ejection fraction (LVEF) and regional wall motion normalize. This checkpoint is indispensable because it confirms resolution of the dominant mechanical phenotype. Its limitation is conceptual: a single global systolic value cannot describe the rate, completeness, or biological breadth of recovery. In the RETAKO registry, recovery at 10 days or later occurred in approximately one quarter of patients and was associated with higher four-year mortality (5). Deep phenotyping has also identified persistent heart failure-type symptoms, impaired exercise capacity, abnormal deformation, increased native T1, and impaired myocardial energetics more than one year after the acute event despite normal LVEF (6).

Persistent symptoms and myocardial abnormalities after apparent systolic recovery are recognized in contemporary TTS reviews (7–9). Rather than recataloguing these findings, this review organizes them around three decisions arising during convalescence: how recovery should be defined, which findings constitute unresolved cardiovascular recovery rather than competing non-cardiovascular risk, and when an additional test could change management. The resulting framework separates mechanical, clinical/functional, and biological/subclinical domains; links testing to a defined question; and makes evidence maturity and causal uncertainty explicit. It is a synthesis for clinical reasoning and prospective evaluation, not a new disease phenotype or a validated follow-up algorithm.

For this State-of-the-Art Review, PubMed/MEDLINE was searched from database inception through July 2026 using combinations of “Takotsubo syndrome” or “stress cardiomyopathy” with terms related to recovery, follow-up, ventricular function, strain, cardiovascular magnetic resonance, myocardial energetics, microvascular function, inflammation, exercise capacity, quality of life, rehabilitation, complications, and prognosis. Crossref was used to verify citation metadata, and the reference lists of major consensus statements, recent reviews, and reviewer-suggested articles were screened. We prioritized peer-reviewed registries, longitudinal cohorts, randomized trials, consensus documents, and systematic reviews that directly informed post-acute recovery or management. Small mechanistic studies were retained when they addressed otherwise unresolved domains and are identified as exploratory; isolated case reports were not used to support general recommendations. This was a narrative rather than systematic review, and no formal risk-of-bias assessment or quantitative synthesis was undertaken.

## 2 How LVEF became the default recovery endpoint

The association between recovery and LVEF normalization arose from the original diagnostic problem. Early Japanese reports described an infarct-like syndrome with marked but reversible ventricular dysfunction and no obstructive culprit lesion (10, 11). Subsequent systematic review and Western case series consolidated transient apical or mid-ventricular dysfunction as a reproducible acute myocardial infarction mimic (12, 13). Reversibility therefore served two purposes: it described a favorable mechanical outcome and helped distinguish TTS from irreversible infarction.

Imaging practice converted this diagnostic feature into an operational endpoint. Ventriculography and echocardiography made regional wall-motion patterns and depressed LVEF immediately visible, while convalescent studies allowed direct comparison with the acute examination. LVEF was familiar, widely available, and readily incorporated into registries and clinical communication. The catecholamine-mediated stunning model further supported abrupt but reversible systolic failure after intense emotional stress (14). Cardiovascular magnetic resonance (CMR) improved tissue characterization and differential diagnosis, particularly against myocarditis and myocardial infarction, while retaining transient ventricular dysfunction as the diagnostic anchor (15).

Position statements and international consensus documents subsequently formalized TTS as a reversible acute heart failure syndrome and embedded reassessment of ventricular function, wall motion, and complications into care pathways (16, 17). The resulting LVEF-centered model was therefore a rational response to the clinical and technological context, not a historical error. The problem emerges only when resolution of the acute mechanical phenotype is equated with full recovery. Global LVEF may normalize despite residual regional mechanics, abnormal tissue composition, altered energetic reserve, or persistent functional limitation. Recovery should therefore be understood as a trajectory in which LVEF is the first checkpoint and recovery tempo provides additional information (5).

## 3 Evidence for persistent and asynchronous post-TTS abnormalities

### 3.1 Mechanical, tissue, and energetic recovery

Recovery trajectory provides information beyond a single convalescent LVEF measurement. In RETAKO, later ventricular recovery was associated with higher long-term all-cause mortality, with excess deaths predominantly attributable to non-cardiovascular causes (5). The study's ≥ 10-day definition should be interpreted as a registry-specific threshold requiring external validation. Delayed recovery is therefore a prognostic marker, not an established modifiable mechanism or a validated trigger for a particular testing strategy.

Residual myocardial mechanical abnormalities can persist after normalization of LVEF and regional wall motion. A prospective echocardiography and CMR study found persistent abnormalities in deformation, contraction timing, relaxation, and tissue characteristics four months after acute TTS despite recovered LVEF and ventricular volumes (18). Long-term phenotyping similarly identified impaired deformation in patients with previous TTS despite normal conventional biomarkers (6). More recent longitudinal strain data suggest that both global and regional deformation may remain abnormal beyond apparent clinical recovery (19). These findings establish a detectable subclinical signal but do not, by themselves, define clinically incomplete recovery; TTS-specific prognostic thresholds and treatment targets for strain have not been established.

Residual tissue and energetic abnormalities have also been reported after TTS. In patients studied more than 12 months after TTS, native T1 remained increased and the phosphocreatine-to-adenosine triphosphate ratio was reduced on phosphorus-31 magnetic resonance spectroscopy (6). At four months, extracellular volume also remained abnormal despite normalization of LVEF and ventricular volumes (18). These findings support persistent tissue and metabolic disturbance, but they arise from small, deeply phenotyped cohorts. A systematic review of CMR prognostication found that strain parameters, right ventricular involvement, and selected CMR-derived models were associated with adverse events, while emphasizing the limited and heterogeneous evidence base (20). CMR is therefore valuable for diagnosis and selected phenotyping, but no single tissue marker yet defines convalescent recovery.

### 3.2 Inflammation and microvascular recovery

Inflammatory and microvascular studies add a second layer of asynchrony. In a multicenter CMR study using ultrasmall superparamagnetic particles of iron oxide, acute TTS was associated with myocardial macrophage activity. The myocardial signal was no longer detectable at five months, whereas selected systemic cytokine and monocyte abnormalities persisted (21). In a separate cohort, a high inflammatory response at discharge was associated with lower follow-up LVEF and a higher risk of cardiac death or heart failure hospitalization (22). These studies identify persistent post-TTS biological signals, but they do not establish that inflammation causes late symptoms or that anti-inflammatory treatment would improve outcomes.

Serial positron emission tomography has provided complementary evidence for vascular recovery. Coronary microvascular function improved after the acute episode but remained regionally abnormal in distal and apical territories months later (23). This pattern is biologically coherent with microvascular dysfunction in TTS, yet routine perfusion positron emission tomography cannot currently be justified for convalescent surveillance. The key contribution of these studies is conceptual: global systolic recovery can precede normalization of tissue, immune, and microvascular signals.

### 3.3 Functional and patient-centered recovery

Persistent dyspnea, fatigue, and exercise intolerance despite normalized resting LVEF provide a direct clinical reason to reassess recovery. Scally et al. documented persistent symptoms and reduced exercise capacity in a selected long-term TTS cohort (6). By contrast, Jabbour et al. identified reduced exercise performance in 40 asymptomatic patients with previous emotion-triggered TTS compared with matched controls (24). These findings distinguish patient-experienced limitation from subclinical physiological impairment; neither cohort establishes their prevalence in all survivors. In selected patients, CPET can characterize circulatory reserve, ventilatory efficiency, and deconditioning when symptoms remain unexplained, but it should answer a defined clinical question rather than serve as routine surveillance.

Patient-reported recovery shows a similar mismatch. Delayed deformation recovery has been associated with poorer physical quality of life (25), while long-term case-control data identify depression, anxiety, chronic stress, and impaired physical and mental health after TTS (26). Stress-associated amygdalar activity has also been linked retrospectively to the subsequent risk and timing of TTS, suggesting that neurobiological vulnerability may precede the acute event (27). This result should not be interpreted as a validated follow-up biomarker, particularly because the cohort was enriched for malignancy. It does, however, reinforce the need to consider psychological and neurocardiac recovery alongside imaging.

Taken together, the literature describes asynchronous persistence of findings across ventricular function, myocardial mechanics, tissue-energetic, inflammatory, microvascular, functional, and patient-reported domains. Table 1 separates each observed signal from its potential use and current boundary; it is not a universal testing panel. Evidence maturity differs substantially. Serial LVEF and regional wall-motion assessment, symptom review, and complication surveillance constitute established clinical practice. Strain, CMR, CPET, and structured patient-reported outcomes are selective adjuncts whose incremental prognostic and management value requires further validation. Positron emission tomography, phosphorus-31 magnetic resonance spectroscopy, and neurobiological imaging remain research assessments.

**Table 1:** Evidence domains across recovery and persistent post-TTS abnormalities.

Recovery domain	Representative measures	Main evidence	Potential use	Current boundary
Tempo and conventional systolic recovery	Days to LV recovery; serial LVEF; regional wall motion	Late recovery (at least 10 days in RETAKO) occurred in 25% and was associated with higher adjusted mortality (5)	Identifies the recovery trajectory and an unresolved acute phenotype	The 10-day threshold is registry-derived and requires external validation
Residual mechanics	Global and regional strain; contraction and relaxation timing; diastolic indices	Deformation abnormalities persisted at four months and within one year after apparent LVEF recovery (18, 19)	May explain symptoms discordant with resting LVEF	TTS-specific prognostic cut-offs and treatment targets are unavailable
Tissue and energetics	Native T1 and extracellular volume; phosphorus-31 MRS; CMR when clinically indicated	Higher native T1 and impaired energetic status persisted beyond 12 months; extracellular volume remained abnormal at four months (6, 18)	Documents persistent post-TTS biological abnormalities beyond conventional systolic recovery	Evidence comes from small selected cohorts; phosphorus-31 MRS remains a research tool
Inflammation and microvascular function	CRP; cytokine and monocyte profiles; USPIO-CMR; PET perfusion	Selected systemic inflammatory signals and regional microvascular abnormalities persisted at approximately five to six months, whereas myocardial USPIO signal resolved by five months (21–23)	Defines candidate mechanisms and risk markers	Causality, incremental value, and treatment responsiveness remain unproven
Functional and patient-centered recovery	CPET; peak VO2; VE/VCO2 slope; symptoms; quality of life; psychological measures	Reduced exercise capacity and psychosocial burden may persist despite normalized LVEF (6, 24–26)	Captures physiological reserve and meaningful recovery	Cohorts are selected; no TTS-specific recovery measure or universal CPET indication is validated

CMR, cardiovascular magnetic resonance; CPET, cardiopulmonary exercise testing; CRP, C-reactive protein; LVEF, left ventricular ejection fraction; LV, left ventricular; MRS, magnetic resonance spectroscopy; PET, positron emission tomography; TTS, takotsubo syndrome; USPIO, ultrasmall superparamagnetic particles of iron oxide; VE/VCO2, minute ventilation/carbon dioxide production. Measures are representative and do not constitute a universal test panel.

Abnormalities detected after TTS are not necessarily abnormalities caused by TTS. As discussed previously, some findings may reflect pre-existing myocardial or systemic susceptibility rather than residual injury from the index episode (28). Pre-event measurements are generally unavailable, limiting causal attribution. Such findings should therefore be described as persistent post-TTS abnormalities unless longitudinal or comparative evidence supports an acquired consequence.

## 4 A multidimensional recovery framework

A useful recovery model should broaden assessment without turning every residual signal into a clinical mandate. Figure 1 organizes recovery into five linked domains: timing; global and regional mechanics; tissue and energetics; vascular and inflammatory regulation; and functional and patient-centered health. These domains are not independent compartments. They describe different views of the same trajectory and may converge in some patients while remaining discordant in others.

**Figure 1:**
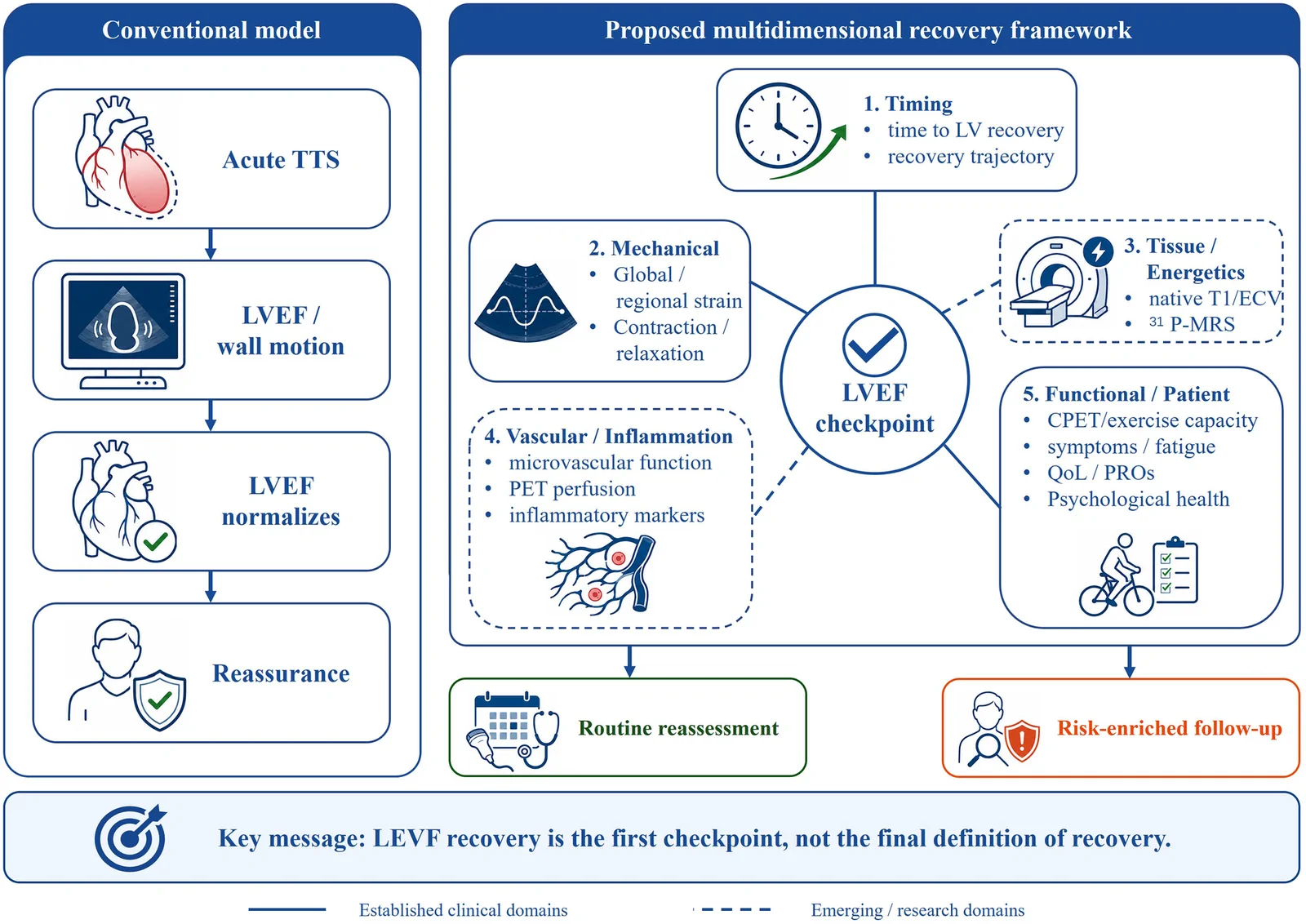
Multidimensional assessment after takotsubo syndrome. The conventional model equates normalization of LVEF and regional wall motion with recovery. The proposed framework retains these measures as the mechanical endpoint and separately considers recovery tempo, residual myocardial mechanics, tissue and energetic signals, vascular and inflammatory regulation, and clinical/functional and patient-centered health. Solid lines indicate established clinical domains; dashed lines indicate emerging or research domains. CMR, cardiovascular magnetic resonance; CPET, cardiopulmonary exercise testing; CRP, C-reactive protein; GLS, global longitudinal strain; LVEF, left ventricular ejection fraction; MRS, magnetic resonance spectroscopy; PET, positron emission tomography; PROs, patient-reported outcomes; QoL, quality of life; TTS, Takotsubo syndrome.

For this review, mechanical recovery denotes normalization of LVEF and regional wall motion, interpreted together with the trajectory of change. Clinical/functional recovery denotes resolution or meaningful improvement of symptoms, return to usual activity, and recovery of patient-reported health; objective exercise capacity is assessed when clinically indicated. Biological/subclinical recovery refers to myocardial deformation, tissue, energetic, inflammatory, or microvascular signals that may remain abnormal after conventional systolic recovery. A biological abnormality alone is not classified as clinically incomplete recovery unless it is linked to symptoms, functional limitation, or clinically meaningful outcomes. Because measurements before TTS are usually unavailable, these signals are described as persistent post-TTS abnormalities rather than sequelae attributable to TTS.

LVEF, regional wall motion, and time to ventricular recovery form the first layer. They confirm reversal of the acute phenotype, identify delayed recovery, and reveal complications that require treatment (5, 17). The second layer addresses residual myocardial biology. Global longitudinal strain may detect subtle mechanical dysfunction, while CMR can reassess ventricular involvement and tissue characteristics when recovery is delayed, the diagnosis remains uncertain, or symptoms exceed echocardiographic findings (18–20). The third layer captures candidate mechanisms, including inflammation and microvascular dysfunction. These measures are hypothesis-generating and should not be converted into routine testing until incremental prognostic value and treatment responsiveness are established. The final layer asks whether the patient has recovered enough to resume ordinary life. Symptoms, physical activity, exercise capacity, psychological health, and quality of life are therefore outcomes rather than optional context. CPET is particularly useful when dyspnea, fatigue, or reduced activity cannot be explained by resting imaging (6, 24). Structured patient-reported outcomes can document whether recovery is meaningful from the patient's perspective, although a TTS-specific validated recovery instrument is not yet available.

The framework is deliberately tiered. Every patient requires clinical reassessment of symptoms, ventricular function, wall motion, complications, and medication indications. Additional tests should answer a defined problem: strain for residual mechanics, CMR for unresolved diagnosis or myocardial characterization, CPET for unexplained functional limitation, and psychological assessment for persistent distress or activity avoidance. Advanced metabolic and neurobiological imaging belongs in research protocols. This hierarchy preserves the clinical value of LVEF while preventing it from becoming the sole definition of recovery.

## 5 Clinical translation: risk-enriched follow-up after TTS

### 5.1 The default post-acute checkpoint

The first post-acute visit should establish whether the diagnosis remains secure, ventricular function and wall motion have recovered, acute complications have resolved, and symptoms are improving. Long-term risk is not negligible: a nationwide cohort found higher risks of death and heart failure hospitalization after TTS than in the background population (29), while a meta-regression estimated annual mortality of 3.5% and recurrence of approximately 1% (30). Much of the excess mortality is non-cardiovascular and reflects comorbidity, so follow-up should not be interpreted as surveillance for TTS recurrence alone.

Risk scores can aid early prognostic thinking but do not define recovery. The InterTAK Prognostic Score integrates age, sex, trigger type, neurological disease, LVEF, and other presentation features to estimate short- and long-term mortality (31). A systematic review similarly identified male sex, physical triggers, malignancy, renal disease, pulmonary disease, and other comorbidities as adverse prognostic features (32). These tools and associations support proportional follow-up; they do not establish fixed schedules or mandatory test panels.

### 5.2 Features that justify risk enrichment

Delayed ventricular recovery is the most direct trigger for closer reassessment. Repeat echocardiography should document the trajectory rather than merely confirm a final value, with strain or CMR selected when recovery remains delayed or symptoms persist (5, 20). Severe initial dysfunction also matters. An admission LVEF of 35% or less was associated with more acute complications and higher long-term major adverse cardiac events in the Takotsubo Italian Network (33).

Trigger and comorbidity refine overall prognosis but should be distinguished from unresolved cardiovascular recovery. In the GEIST registry, physically triggered TTS carried higher in-hospital event rates and long-term mortality than emotionally triggered disease (34). Acute pulmonary triggers were independently associated with worse long-term mortality in the International Takotsubo Registry (35). Neurological disorders define a high-risk phenotype (36), malignancy independently predicts long-term mortality (37), and men experience higher rates of shock, arrhythmia, and mortality than women despite representing a minority of cases (38). These features often mark competing illness and the need for multidisciplinary or comorbidity-directed care. In isolation, they do not establish that advanced cardiac testing will provide incremental value.

Acute physiology should guide the content of follow-up. Right ventricular involvement identifies a phenotype with a more complicated presentation and adverse outcomes (39). Cardiogenic shock is heterogeneous, and its mechanism may include pump failure, right ventricular dysfunction, left ventricular outflow tract obstruction (LVOTO), or acute mitral regurgitation (40). In the largest recent RETAKO analysis, LVOTO was identified in 155 of 1,580 patients (9.8%) and was associated with apical ballooning, systolic anterior motion, moderate-to-severe mitral regurgitation, and more cardiogenic shock, but not with higher in-hospital or long-term mortality; obstruction resolved in 93.4% of patients with follow-up echocardiography (41). These data support mechanism-specific acute management and documentation of resolution, but not routine long-term advanced testing solely because LVOTO occurred.

Thromboembolic risk requires a separate pathway. Intraventricular thrombus or embolism occurred in 3.3% of patients in the International Takotsubo Registry and was associated with apical ballooning, severe LVEF reduction, vascular disease, and leukocytosis (42). RETAKO reported ventricular thrombus in 2.8% of patients, with higher inflammatory burden and late gadolinium enhancement as independent predictors and markedly higher in-hospital embolic events (43). These data support targeted repeat imaging and anticoagulation review in high-risk patients, not routine anticoagulation for all survivors.

Persistent symptoms can reveal risk that was not apparent at discharge. Dyspnea, fatigue, chest discomfort, reduced exercise tolerance, or poor quality of life should prompt reassessment even when LVEF has normalized. CRP at discharge may add prognostic information to the InterTAK score, but the supporting evidence remains retrospective and should not convert an inflammatory marker into a treatment target (44). CMR, strain, CPET, and structured patient-reported outcomes can then be selected according to the unresolved clinical question (20, 24, 45). Figure 2 shows the overall pathway, whereas Table 2 links risk-enrichment features to question-driven reassessment and explicitly avoids prescribing a fixed schedule.

**Figure 2:**
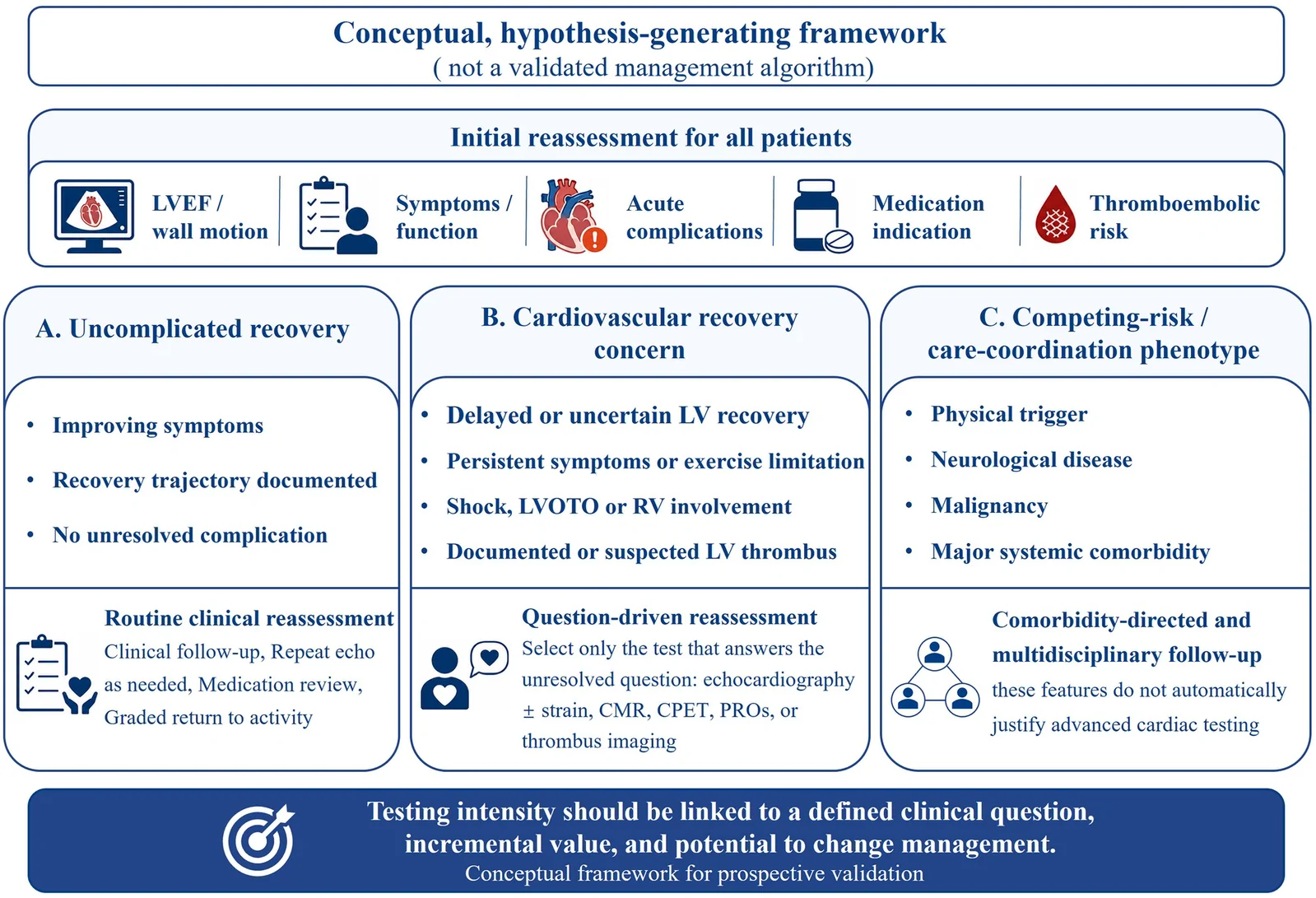
Conceptual, question-driven follow-up framework after takotsubo syndrome. Initial reassessment is common to all patients. Unresolved cardiovascular recovery concerns—delayed or uncertain left ventricular recovery, persistent symptoms or exercise limitation, severe acute cardiovascular complications, and documented or suspected ventricular thrombus—may justify targeted reassessment selected to answer a defined clinical question. By contrast, physical triggers, neurological disease, malignancy, and major systemic comorbidity primarily indicate competing risk and a need for coordinated multidisciplinary care; they do not automatically justify advanced cardiac testing. The figure is hypothesis-generating and is not a validated management algorithm or fixed test panel. CMR, cardiovascular magnetic resonance; CPET, cardiopulmonary exercise testing; LV, left ventricular; LVEF, left ventricular ejection fraction; LVOTO, left ventricular outflow tract obstruction; PROs, patient-reported outcomes; RV, right ventricular; TTS, Takotsubo syndrome.

**Table 2:** Cardiovascular recovery concerns and competing-risk features after takotsubo syndrome.

Cardiovascular recovery concern/competing-risk feature	Question-driven reassessment	Evidence basis and boundary
Delayed or absent early LV recovery	Has LVEF and regional wall motion normalized, and have acute complications resolved? Repeat echocardiography; use strain or CMR only for a defined unresolved question	Recovery tempo is prognostic, but the 10-day RETAKO threshold and test-guided care require external validation (5, 20)
Persistent symptoms despite normalized LVEF	Are symptoms related to residual mechanics, deconditioning, pulmonary disease, or psychological distress? Use structured symptom or quality-of-life assessment; select CPET or imaging accordingly	Small selected cohorts support residual functional limitation; outcome-linked validation of this testing strategy is lacking (6, 24–26, 45)
Severe initial LV dysfunction, shock, LVOTO, or right ventricular involvement	Which acute mechanism remains relevant? Reassess biventricular function, wall motion, mitral regurgitation, and LVOTO according to the index complication	These features identify higher acute or long-term risk, but no validated post-discharge schedule exists (33, 39, 40, 46)
Apical ballooning with severe LV dysfunction or documented or suspected LV thrombus	Has thrombus resolved and does embolic risk still exceed bleeding risk? Use early repeat imaging and individualized anticoagulation review	Thrombus is uncommon but clinically important; evidence supports targeted rather than universal anticoagulation (42, 43, 47)
Physical or neurological trigger; male sex	Is risk driven by the precipitating illness or comorbidity? Coordinate trigger-specific and multidisciplinary care; do not order advanced testing automatically	Associations identify higher-risk groups but do not prove benefit from more intensive cardiac testing (34–38)
Malignancy or major systemic comorbidity	Which competing risks dominate prognosis? Prioritize comorbidity-directed follow-up and cardio-oncology or multidisciplinary input when relevant	Non-cardiovascular risk may dominate long-term outcome (37, 48)
Elevated CRP at discharge	Does the signal reflect unresolved TTS, infection, malignancy, or another systemic process? Integrate CRP with established clinical risk and avoid escalation on CRP alone	Retrospective studies support prognostic association, not serial CRP surveillance or inflammation-directed treatment (22, 44)

CMR, cardiovascular magnetic resonance; CPET, cardiopulmonary exercise testing; CRP, C-reactive protein; LVEF, left ventricular ejection fraction; LV, left ventricular; LVOTO, left ventricular outflow tract obstruction; TTS, Takotsubo syndrome. Most associations are observational. The table organizes clinical questions and does not prescribe a validated schedule or management algorithm.

## 6 Management implications: From reassurance to rehabilitation

A recovery-based approach changes the purpose of post-acute management. Reassurance remains appropriate because ventricular function often improves rapidly, but it should be paired with scheduled reassessment, medication review, complication surveillance, and a graded return to activity. The therapeutic question is not how to treat every residual biomarker. It is how to address the abnormality or limitation that remains clinically relevant.

Pharmacological care should remain indication-driven. A network meta-analysis found no convincing evidence that beta-blockers, angiotensin-converting enzyme inhibitors, angiotensin receptor blockers, or their combination prevent recurrence (49). A Bayesian network meta-analysis and a large GEIST analysis associated beta-blocker therapy with lower mortality, but these findings are derived predominantly from non-randomized data and do not establish TTS-specific efficacy (50, 51). The *β*-Tako randomized trial was designed to compare beta-blocker therapy with no beta-blocker therapy in 200 patients (52). Its primary endpoint is the echocardiographic wall-motion score index at 7 days, with clinical outcomes assessed at 1 year. The published report describes the trial design rather than treatment results; it therefore addresses the evidence gap without establishing efficacy. Long-term antiplatelet therapy also lacks a TTS-specific rationale in the absence of coronary or other vascular indications; a meta-analysis found no long-term benefit and raised concern about harm, although all included studies were retrospective (53). Medication should therefore be guided by residual ventricular dysfunction, hypertension, coronary disease, arrhythmia, LVOTO, thromboembolic risk, or another established indication.

Documented LV thrombus and thrombotic risk without demonstrated thrombus require separate decisions. For documented thrombus, anticoagulation and repeat imaging are directed at preventing embolism and confirming resolution. In GEIST, thrombi resolved after 3 months of oral anticoagulation, supporting reassessment around this interval rather than a universally fixed duration (47). Without demonstrated thrombus, empirical anticoagulation may be considered in selected patients with extensive apical akinesis and severe LV dysfunction (17). This approach rests on observational evidence and expert judgement, not randomized proof of net benefit. More recent RETAKO data reinforce the importance of balancing embolic and bleeding risk (43). Embolic risk must be weighed against bleeding risk, with treatment reconsidered as ventricular function and thrombotic risk evolve.

Randomized evidence provides direct support for targeting selected recovery domains. In a multicenter trial involving 76 patients, 12 weeks of exercise training or cognitive behavioral therapy improved myocardial energetics and exercise performance compared with standard care. Neither intervention demonstrated improvement in global longitudinal strain or heart failure symptom burden (54). These findings support domain-specific benefits, not comprehensive recovery or reduced clinical-event risk. Participants were recruited early after TTS, so efficacy in established late symptomatic syndromes remains uncertain. Rehabilitation should be individualized according to clinical stability, functional limitation, psychological needs, and patient preference rather than prescribed universally. Observational reports of low rehabilitation uptake identify an implementation issue but do not establish population-wide effectiveness (55).

Psychological recovery belongs in the same pathway. Depression, anxiety, chronic stress, sleep disturbance, and post-event fear may restrict activity and amplify symptom burden (26). Screening is most useful when it leads to an intervention, such as integrated cardiac rehabilitation, cognitive behavioral therapy, or specialist mental health care. The goal is not to psychologize persistent cardiopulmonary symptoms, but to identify modifiable barriers while mechanical and functional causes are assessed in parallel.

Recurrence prevention remains uncertain. Population-based follow-up confirms that recurrence affects a minority of patients and that age, male sex, diabetes, pulmonary disease, and chronic kidney disease are associated with the combined risk of death or recurrence (56). A systematic review estimated recurrence at 1%–3.5% per year and found inconsistent predictors (57). Patients should receive clear advice about recurrent symptoms and prompt reassessment, but neither chronic medication nor repeated advanced imaging can currently be justified solely to prevent recurrence.

## 7 Controversies and research priorities

The central controversy is not whether LVEF matters. It is whether a normal LVEF should close the recovery assessment. The evidence supports a middle position: LVEF is necessary, recovery tempo is prognostically informative, and selected patients have residual biological or functional abnormalities. What remains uncertain is whether these abnormalities improve risk prediction, identify treatable mechanisms, or change outcomes beyond careful clinical follow-up.

The first priority is temporal definition. Studies use heterogeneous follow-up intervals and often classify recovery at a single visit. Future cohorts should distinguish acute mechanical recovery, early post-discharge stabilization, intermediate myocardial recovery, and late functional recovery. Serial measurements should be anchored to clinically meaningful outcomes rather than to imaging change alone. Age also needs explicit modeling because younger patients have more non-apical phenotypes and in-hospital complications, whereas older patients have substantially higher short- and long-term mortality (58).

The second priority is a core recovery set. A pragmatic minimum should combine LVEF and wall motion with symptoms, return to activity, and a validated generic or heart failure patient-reported measure. Strain, CMR, CPET, inflammatory biomarkers, and microvascular imaging should be tested for incremental value against this core. The optimal set must be feasible across centers and sensitive to change. Without such standardization, multidimensional recovery risks becoming a collection of interesting but non-comparable signals.

The third priority is to distinguish abnormalities persisting after TTS from abnormalities attributable to TTS. Prospective studies should combine serial within-patient assessment with appropriately matched non-TTS controls, including comparable comorbidity and precipitating illness where feasible. Pre-event imaging or functional measurements should be incorporated when available. These designs can help separate acquired consequences, pre-existing susceptibility, and their interaction, although causal interpretation will still require attention to confounding.

The fourth priority is phenotype-stratified intervention. Recovery trajectories should be compared by trigger, sex, age, neurological disease, malignancy, shock mechanism, ventricular involvement, and baseline LVEF. Long-term follow-up from Vienna illustrates that non-cardiovascular mortality, particularly malignancy and renal disease, can dominate prognosis (48). Trials should therefore separate TTS-specific outcomes from competing comorbidity and test whether exercise, cognitive behavioral therapy, complication-directed care, or phenotype-guided medication improves patient-centered recovery.

Finally, implementation must remain proportional. A recovery framework will be useful only if it identifies who benefits from additional assessment and which test changes management. Table 3 therefore separates validation of the risk-enrichment strategy from studies of recovery mechanisms and interventions. Prospective evaluation should compare the framework with conventional follow-up using mortality, heart failure hospitalization, recurrent TTS, exercise capacity, quality of life, return to usual activity, and resource use. Adjunctive tests should be evaluated against the pragmatic core assessment for incremental prediction, management changes, patient-centered outcomes, testing burden, and cost.

**Table 3:** Research priorities for multidimensional recovery after takotsubo syndrome.

Priority	Key question	Recommended design or endpoint	Decision boundary
Define temporal recovery stages	When should each recovery domain be assessed?	Prospective multicenter cohorts with prespecified acute, early, intermediate, and late windows anchored to the index event	Timing thresholds must be linked to outcomes before clinical adoption
Establish a core recovery set	Which minimum measures add value beyond LVEF?	LVEF, wall motion, symptoms, return to activity, and patient-reported outcomes; test incremental value of strain, CMR, and CPET	The set must be feasible, responsive to change, and externally validated
Validate risk-enriched follow-up	Does the proposed strategy improve care compared with conventional follow-up?	Pragmatic prospective comparison using clinical events, exercise capacity, quality of life, return to activity, and resource use	Benefit, burden, and cost must be evaluated before implementation
Stratify by phenotype and comorbidity	Do trajectories differ by trigger, sex, age, neurological disease, shock mechanism, or comorbidity?	Registry-linked prospective cohorts with prespecified interaction tests and competing-risk models	Avoid *post hoc* subgroup claims and attribution of comorbidity-driven events to TTS
Link mechanisms to outcomes	Do inflammation, microvascular dysfunction, or energetic impairment explain symptoms or events?	Longitudinal multimodality assessment in the same cohort; mediation analysis and mechanistic intervention studies	Association should not be interpreted as causality
Test recovery-directed interventions	Which interventions improve function and quality of life?	Randomized trials of exercise, cognitive behavioral therapy, complication-directed care, or phenotype-guided pharmacology	Use domain-specific outcomes and monitor harms

CMR, cardiovascular magnetic resonance; CPET, cardiopulmonary exercise testing; LVEF, left ventricular ejection fraction; TTS, Takotsubo syndrome. Priorities are the authors’ synthesis and not formal consensus recommendations.

## 8 Conclusion

Recovery after Takotsubo syndrome is best assessed as a trajectory rather than a binary imaging endpoint. Normalization of LVEF and regional wall motion confirms mechanical recovery, whereas persistent symptoms or functional limitation identifies clinically incomplete recovery. Residual strain, tissue, energetic, microvascular, or inflammatory signals should be described as biological/subclinical abnormalities unless they are linked to symptoms, function, or outcomes. This distinction avoids converting a detectable abnormality into a diagnosis or an indication for routine testing.

Follow-up should be proportional and question-driven. Uncomplicated recovery warrants routine clinical and echocardiographic reassessment; unresolved cardiovascular recovery concerns may justify targeted testing, whereas malignancy, neurological disease, physical triggers, and major systemic comorbidity primarily call for coordinated care rather than automatic advanced cardiac investigation. The proposed framework is a hypothesis-generating structure for prospective validation, not a management algorithm.
